# Evaluation of the effect of tofacitinib on measured glomerular filtration rate in patients with active rheumatoid arthritis: results from a randomised controlled trial

**DOI:** 10.1186/s13075-015-0612-7

**Published:** 2015-04-06

**Authors:** Joel M Kremer, Alan J Kivitz, Jesus A Simon-Campos, Evgeny L Nasonov, Hans-Peter Tony, Soo-Kon Lee, Bonnie Vlahos, Constance Hammond, Jack Bukowski, Huihua Li, Seth L Schulman, Susan Raber, Andrea Zuckerman, John D Isaacs

**Affiliations:** Albany Medical College and The Center for Rheumatology, Albany, NY USA; Altoona Center for Clinical Research, Duncansville, PA USA; Hospital CEM/BIOCEM, Merida, Mexico; Nasonova State Institute of Rheumatology, Moscow, Russia; University Hospital Würzburg, Würzburg, Germany; Yonsei University College of Medicine, Seoul, South Korea; Pfizer Inc, Collegeville, PA USA; Pfizer Inc, San Diego, CA USA; Pfizer Inc, Groton, CT USA; National Institute for Health Research Newcastle Biomedical Research Centre, Newcastle upon Tyne Hospitals NHS Foundation Trust and Newcastle University, Newcastle-upon-Tyne, UK

## Abstract

**Introduction:**

Tofacitinib is an oral Janus kinase inhibitor for the treatment of rheumatoid arthritis (RA). During the clinical development programme, increases in mean serum creatinine (SCr) of approximately 0.07 mg/dL and 0.08 mg/dL were observed which plateaued early. This study assessed changes in measured glomerular filtration rate (mGFR) with tofacitinib relative to placebo in patients with active RA.

**Methods:**

This was a randomised, placebo-controlled, Phase 1 study (NCT01484561). Patients were aged ≥18 years with active RA. Patients were randomised 2:1 to oral tofacitinib 10 mg twice daily (BID) in Period 1 then placebo BID in Period 2 (tofacitinib → placebo); or oral placebo BID in both Periods (placebo → placebo). Change in mGFR was evaluated by iohexol serum clearance at four time points (run-in, pre-dose in Period 1, Period 1 end, and Period 2 end). The primary endpoint was the change in mGFR from baseline to Period 1 end. Secondary endpoints included: change in mGFR at other time points; change in estimated GFR (eGFR; Cockcroft–Gault equation) and SCr; efficacy; and safety.

**Results:**

148 patients were randomised to tofacitinib → placebo (N = 97) or placebo → placebo (N = 51). Baseline characteristics were similar between groups. A reduction of 8% (90% confidence interval [CI]: 2%, 14%) from baseline in adjusted geometric mean mGFR was observed during tofacitinib treatment in Period 1 vs placebo. During Period 2, mean mGFR returned towards baseline during placebo treatment, and there was no difference between the two treatment groups at the end of the study – ratio (tofacitinib → placebo/placebo → placebo) of adjusted geometric mean fold change of mGFR was 1.04 (90% CI: 0.97, 1.11). Post-hoc analyses, focussed on mGFR variability in placebo → placebo patients, were consistent with this conclusion. At study end, similar results were observed for eGFR and SCr. Clinical efficacy and safety were consistent with prior studies.

**Conclusion:**

Increases in mean SCr and decreases in eGFR in tofacitinib-treated patients with RA may occur in parallel with decreases in mean mGFR; mGFR returned towards baseline after tofacitinib discontinuation, with no significant difference vs placebo, even after post-hoc analyses. Safety monitoring will continue in ongoing and future clinical studies and routine pharmacovigilance.

**Trial registration:**

Clinicaltrials.gov NCT01484561. Registered 30 November 2011.

**Electronic supplementary material:**

The online version of this article (doi:10.1186/s13075-015-0612-7) contains supplementary material, which is available to authorized users.

## Introduction

Tofacitinib is an oral Janus kinase (JAK) inhibitor for the treatment of rheumatoid arthritis (RA). In six phase-3 randomised controlled trials, tofacitinib 5 and 10 mg twice daily (BID) as monotherapy or in combination with nonbiologic disease-modifying antirheumatic drugs (DMARDs) were effective in reducing the signs and symptoms of RA, improving physical function, patient-reported outcomes, and structural preservation, and had a consistent safety profile [1-6].

Tofacitinib treatment in pooled RA phase-3 studies over 12 months resulted in mean increases from baseline in serum creatinine (SCr) levels [7]. Increases occurred predominantly within the first 3 months. Mean SCr increases at month 3 were 0.07 and 0.08 mg/dL for tofacitinib 5- and 10-mg BID doses, respectively, compared with 0.04 mg/dL and 0.06 mg/dL in the placebo and adalimumab groups, respectively [7]. Further analyses suggested that effects on mean SCr plateaued, remained within normal limits, were reversible and did not appear to be associated with acute renal failure (ARF) or progressive worsening of renal function in the long-term extension (LTE) studies [7]. The mechanism behind these SCr changes with tofacitinib treatment is unknown; however, it may involve tofacitinib-induced changes in inflammation and creatine kinase [7].

General toxicology preclinical investigations had demonstrated that tofacitinib was not nephrotoxic in rats and monkeys [8,9]. In a placebo-controlled, healthy volunteer study, tofacitinib treatment (up to 15 mg BID for 14 days) had no effect on glomerular filtration rate (GFR), effective renal plasma flow or creatinine clearance [10]. Additionally, based upon a study of metformin in healthy volunteers, tofacitinib does not appear to interfere with tubular transport of creatinine [11,12].

Within the literature, it is important to recognise that changes in SCr and measured GFR (mGFR) do not always correlate [13-16]. As SCr is derived from muscle creatine, many factors affect SCr independently of GFR and include, but are not limited to: age, gender, race, medications, diet, illness, muscle mass and muscle turnover [13-16]. Patients with RA have reduced physical activity and muscle mass, and present with various co-morbidities, as well as increased inflammation [17-20]. Indeed, a prior analysis showed that the greater the baseline C-reactive protein (CRP) and the greater the change in CRP with tofacitinib treatment, the greater the change in SCr [7]. Therefore, the interpretation of SCr levels is complex in this population, and these factors need to be taken into consideration when estimating GFR in patients with RA.

While no effect of tofacitinib on mGFR was seen in healthy volunteers, the interaction between inflammation and potential metabolic mechanisms influencing SCr suggests that measurement of GFR in patients with RA is necessary to fully understand the impact of tofacitinib on renal function. Therefore, the objectives of this study were to determine whether tofacitinib induced changes in mGFR relative to placebo in this population of patients with active RA, and the extent to which these changes, if any, were reversible after discontinuation of tofacitinib treatment.

## Methods

### Patients

Eligible participants were ≥18 years of age with a diagnosis of RA based on the American College of Rheumatology (ACR) 1987 Revised Criteria for Classification of RA [21]. Patients must have had an inadequate response to at least one DMARD (non-biologic or biologic) due to lack of efficacy or intolerance. Other key inclusion criteria included active RA defined by ≥4 tender/painful joints and ≥4 swollen joints, and either erythrocyte sedimentation rate (Westergren method) >28 mm/h or CRP >7 mg/L. Patients were excluded if they had an estimated GFR (eGFR) <40 mL/min (Cockcroft-Gault calculation).

Biologic DMARDs, potent immunosuppressive agents (for example, cyclosporine, azathioprine), injectable gold, penicillamine, intravenous (IV), intramuscular and intra-articular corticosteroids were prohibited. The patient was permitted (but was not required) to continue on background non-biologic DMARDs.

### Study design

This was a phase-1, randomised, placebo-controlled, parallel-group, two-period study (A3921152; NCT01484561). The study was conducted at 23 study centres (one in the Czech Republic, Korea and Mexico; three in Germany; four in Poland, Russia and Spain; five in the USA). There were two study centres in Germany that received the study drug, but did not randomise patients. Patients were randomised 2:1 to one of two fixed sequences, each consisting of two study periods: patients received oral tofacitinib 10 mg BID in period 1 and oral placebo BID in period 2 (tofacitinib → placebo) or patients received oral placebo BID in periods 1 and 2 (placebo → placebo). Randomisation was achieved using an interactive voice response system - an automated web/telephone randomisation system containing the randomisation schedule. Protocol amendments are listed in Additional file 1.

Data from phase-2 and phase-3 studies suggested a greater increase in SCr with the tofacitinib 10-mg BID dose versus the tofacitinib 5-mg BID dose; therefore, in order to increase the probability of observing any potential effects on mGFR, the tofacitinib 10-mg BID dose was selected for investigation in this study. From population modelling analysis of the phase-2 study results, steady-state SCr increases were predicted to be achieved in approximately 6 weeks in a typical patient with RA. Therefore, the duration of tofacitinib treatment in this study was chosen to be 6 weeks (through day 43 in period 1). In a phase-2 study, modelling analysis predicted that after cessation of tofacitinib administration, the 90% upper confidence limit of the mean increase in SCr fell below 10% of pre-treatment baseline within 2 to 6 weeks. Therefore, to evaluate reversibility of the increase in SCr in this study, renal function was to be evaluated 4 weeks after the end of tofacitinib treatment (day 29 in period 2). The selection of the tofacitinib 10-mg BID dose was based on the prior phase-2 and phase-3 studies of tofacitinib in RA.

In period 1, patients received the study drug (tofacitinib or placebo) in the evening of day 1 through to at least the morning of day 43 (up to the morning of day 50). Period 2 immediately followed the end of period 1. In period 2, patients received placebo BID in the evening of day 1 through to at least the morning of day 29 (up to the morning of day 36). In period 1, the patient, the investigator, the site staff and the sponsor were blinded to individual patients’ treatment assignment. In period 2, only patients remained blinded. For the duration of the study, the patients, investigator, the site staff and sponsor remained blinded to the assigned treatment sequence. The approximate total duration of treatment with study drug (tofacitinib or placebo) in periods 1 and 2 was at least 10 weeks (up to 12 weeks). Patients who completed at least period 1 through day 43 in this study were given the option (if they were eligible for enrollment) to enrol in an open-label LTE study A3921024 (NCT00413699).

The study was conducted in compliance with the provisions of the Declaration of Helsinki Good Clinical Practice guidelines of the International Conference on Harmonisation and relevant local country regulations. All patients provided written informed consent. The final protocol, amendments and consent documentation were reviewed and approved by the Institutional Review Board and/or Independent Ethics Committee at each participating centre (Additional file 1).

### Study endpoints and assessments

The study was exploratory in nature and was designed with a primary objective of estimating the ratio (tofacitinib → placebo/placebo → placebo) in the adjusted geometric mean fold-change of mGFR from baseline to period 1 end. Secondary endpoints included: change in mGFR following tofacitinib withdrawal; changes in eGFR (Cockcroft-Gault equation) and SCr with tofacitinib treatment relative to placebo, and evaluation of the efficacy and safety of tofacitinib in patients with active RA.

Each patient underwent mGFR evaluations by determining iohexol serum clearance at four pre-scheduled time points: during the run-in period (between day 14 and day 2), pre-dose on day 1 of period 1, post-dose on the last day of period 1 and post-dose on the last day of period 2. Additionally, SCr levels were obtained at screening, pre-dose on day 1 of period 1, and on the last day of period 1 and of period 2. eGFR was calculated (using the Cockcroft-Gault equation) at both screening and pre-dose on day 1 of period 1 by the site, then at all visits during treatment with the study drug by the sponsor. Iohexol serum clearance was determined after an IV bolus injection of non-radioactive iohexol followed by serial blood sampling. Further details on the iohexol serum clearance procedure can be found in Additional file 1.

Efficacy was assessed by ACR response rates of 20%, 50% and 70% (ACR20/50/70); disease activity score in 28 joints (DAS28)-3(CRP)/DAS28-4(CRP); and their components. The incidence and severity of all adverse events (AEs) were recorded; clinical laboratory tests, vital signs and physical examinations were performed at scheduled visits.

### Statistical analyses

Details on sample size determination can be found in Additional file 1. Statistical analyses focused on point estimations and confidence intervals (CIs). No formal hypothesis test was performed; *P*-values are considered descriptive statistics. All analyses were performed on the full analysis set (FAS) unless otherwise stated – defined as all randomised patients who received ≥1 dose of study drug (tofacitinib or placebo). Additionally, analysis of GFR data was performed on the per-protocol analysis set (PPAS) - defined as a subset of patients in the FAS, who completed the treatment period and who had no major protocol deviations that may impact renal function assessments.

mGFR was determined as iohexol serum clearance using a non-linear mixed-effects modelling approach (NONMEM version 7.2) of the iohexol serum concentration-time data. Serial blood sample collection was designed to maximise precision of the estimate, assuming a two-compartment model based on a prior study and literature reports [10,22,23]. Iohexol clearance values were also normalised by body surface area.

Baseline mGFR was calculated as the mean of the values obtained in the run-in period and pre-dose on day 1 of period 1. Following natural log transformation, an analysis of covariance (ANCOVA) model was used to analyse the mGFR change from baseline with treatment and baseline mGFR (natural log-transformed) as covariates. For change from period 1 end to period 2 end, the ANCOVA model included treatment and the mGFR collected at period 1 end (on log scale) as covariates. The treatment difference and two-sided 90% CI on the log-transformed scale were then back-transformed to derive the treatment ratios for the geometric mean fold-changes. Additional sensitivity analysis using a linear mixed-effects model was performed with mGFR value (on log scale) as a response variable, treatment group, visit and treatment group by visit interaction as fixed effects and patients as random effect. The eGFR and SCr data were analysed in a similar manner.

To assess the robustness of our findings on the mGFR changes, post-hoc ANCOVA was performed to exclude patients who had an outlier mGFR result. An outlier mGFR result was defined as the difference between mGFR and eGFR falling outside the 95% limits of agreement between the two methods of measurement. Distribution of mGFR changes were graphically examined using box and whisker plots.

For ACR variables, the normal approximation for the difference in the binomial proportions was performed at the end of each period. Response rate was calculated for the data as is (no imputation) and using the last observation carried forward for skipped components and the non-responder imputation after early withdrawals.

DAS28-3(CRP)/DAS28-4(CRP) was expressed as change from baseline. The analysis was done using the ANCOVA model including treatment and baseline as covariate. The endpoint was also analysed by a linear mixed-effects model with actual value as a response variable, treatment group, visit and treatment group by visit interaction as fixed effects and patients as random effect.

## Results

### Patients

**Figure 1:**
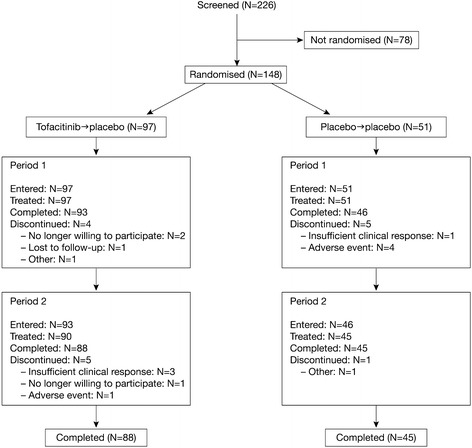
Consolidated standards of reporting trials (CONSORT) diagram.

A total of 148 patients were randomised to treatment (tofacitinib → placebo, N = 97; placebo → placebo, N = 51) and received at least one dose of study medication. Of these, 133 patients (89.9%) completed the study (tofacitinib → placebo, N = 88; placebo → placebo, N = 45). All 148 patients were included in the FAS and were evaluated for safety. Patient disposition is summarised in Figure 1.

The majority of patients were female (75.0%) and white (93.9%), with a mean age of 50.3 years (range 22 to 77 years). RA disease duration ranged from 0.3 to 37 years. Patient demographics and baseline characteristics are summarised in Table 1.

**Table 1:** **Summary of baseline demographics and disease characteristics (full analysis set)**

**Characteristic**	**Tofacitinib** → **placebo group**	**Placebo** → **placebo group**
	**(N = 97)**	**(N = 51)**
Mean (SD) age, years	51.86 (12.68)	47.25 (11.84)
Sex, n (%)		
Male	22 (22.7)	15 (29.4)
Female	75 (77.3)	36 (70.6)
Race, n (%)		
White	90 (92.8)	49 (96.1)
Asian	3 (3.1)	1 (2.0)
Black	3 (3.1)	0
Other	1 (1.0)	1 (2.0)
Mean (SD) body mass index, kg/m^2^	28.71 (6.16)	28.11 (7.13)
Mean (SD) duration of rheumatoid arthritis	8.67 (7.49)	8.25 (7.94)
Prior corticosteroid use, n (%)		
Yes	50 (51.5)	21 (41.2)
No	47 (48.5)	30 (58.8)
Prior DMARD use, n (%)		
Yes	94 (96.9)	47 (92.2)
No	3 (3.1)	4 (7.8)
Prior NSAID use, n (%)		
Yes	73 (75.3)	35 (68.6)
No	24 (24.7)	16 (31.4)
Prior methotrexate use, n (%)		
Yes	87 (89.7)	47 (92.2)
No	10 (10.3)	4 (7.8)
Mean (SD) erythrocyte sedimentation rate, mm/h	45.34 (17.43)	47.08 (23.83)
Mean (SD) C-reactive protein, mg/L	18.37 (22.57)	15.66 (22.55)
Rheumatoid factor, n (%)		
Positive	69 (71.1)	34 (66.7)
Negative	28 (28.9)	17 (33.3)
Mean (SD) DAS28-3(CRP)	5.27 (0.95)	5.31 (0.81)
Mean (SD) DAS28-4(CRP)	5.50 (1.05)	5.56 (0.93)
Mean (SD) Patient Assessment of Arthritis Pain	53.98 (22.77)	57.45 (21.04)
Mean (SD) Patient Global Assessment of Arthritis	56.13 (23.78)	58.67 (21.70)
Mean (SD) Physician Global Assessment of Arthritis	57.24 (17.83)	55.22 (17.11)
Mean (SD) tender/painful joint counts	20.41 (11.63)	23.43 (13.34)
Mean (SD) swollen joint counts	12.48 (6.76)	12.67 (6.91)
Mean (SD) HAQ-DI	1.49 (0.65)	1.41 (0.69)

DAS28, disease activity score in 28 joints; DMARD, disease-modifying antirheumatic drug; HAQ-DI, health assessment questionnaire-disability index; NSAID, non-steroidal anti-inflammatory drug; SD, standard deviation.

### Renal endpoints

#### mGFR

Baseline mGFR was comparable between the two treatment groups (Table 2). In the tofacitinib → placebo group, median mGFR decreased by 6.46 mL/min/1.73 m^2^ from baseline to period 1 end (Table 2). Following tofacitinib withdrawal in period 2, median mGFR increased by 1.68 mL/min/1.73 m^2^ compared with period 1. In the placebo → placebo group, there was little change in mGFR at period 1 end; however, at the end of period 2, median mGFR decreased by 3.25 mL/min/1.73 m^2^ (Table 2). Overall, at period 2 end compared with baseline, median mGFR decreased by 3.41 mL/min/1.73 m^2^ in the tofacitinib → placebo group and by 3.88 mL/min/1.73 m^2^ in the placebo → placebo group (Table 2).

**Table 2:** **Descriptive statistics of change in mGFR, eGFR (Cockcroft-Gault) and serum creatinine (full analysis set)**

	**mGFR (mL/min/1.73 m** ^**2**^ **)**		**eGFR (Cockcroft-Gault) (mL/min/1.73 m** ^**2**^ **)**		**Serum creatinine (mg/dL)**	
	**Tofacitinib** → **placebo group**	**Placebo** → **placebo group**	**Tofacitinib** → **placebo group**	**Placebo** → **placebo group**	**Tofacitinib** → **placebo group**	**Placebo** → **placebo group**
**Baseline**						
Number	97	51	97	51	97	51
Mean (SD)	88.98 (18.79)	89.67 (21.33)	104.19 (28.42)	111.30 (26.91)	0.77 (0.15)	0.76 (0.17)
Median (Q1, Q3)	87.98 (75.76, 99.98)	86.99 (75.17, 98.20)	97.22 (82.43, 125.57)	107.22 (91.47, 134.89)	0.76 (0.67, 0.85)	0.75 (0.64, 0.84)
**Change at end of period 1 from baseline**						
Number	91	46	92	46	92	46
Mean (SD)	−6.73 (16.44)	−0.50 (17.34)	−3.59 (10.31)	1.28 (11.48)	0.03 (0.07)	−0.00 (0.07)
Median (Q1, Q3)	−6.46 (−11.85, 2.14)	0.82 (−5.48, 6.79)	−3.36 (−9.18, 2.86)	2.30 (−5.71, 6.14)	0.02 (−0.02, 0.08)	−0.01 (−0.05, 0.04)
**Change at end of period 2 from baseline**						
Number	86	45	87	44	87	44
Mean (SD)	−3.02 (17.55)	−5.96 (20.66)	0.03 (10.15)	0.48 (9.54)	0.00 (0.07)	−0.00 (0.05)
Median (Q1, Q3)	−3.41 (−8.55, 3.65)	−3.88 (−9.13, 7.58)	−0.32 (−6.05, 5.74)	0.18 (−3.81, 4.28)	0.00 (−0.05, 0.05)	0.00 (−0.03, 0.03)
**Change at end of period 2 from end of period 1**						
Number	86	45	87	44	87	44
Mean (SD)	2.92 (19.02)	−5.41 (16.65)	3.71 (11.95)	−0.97 (10.06)	−0.03 (0.08)	0.00 (0.07)
Median (Q1, Q3)	1.68 (−3.60, 8.62)	−3.25 (−12.24, 2.29)	2.94 (−4.45, 10.31)	−2.07 (−7.29, 6.09)	−0.02 (−0.07, 0.03)	0.01 (−0.04, 0.05)

eGFR, estimated glomerular filtration rate; mGFR, measured glomerular filtration rate; Q1, 25th percentile; Q3, 75th percentile; SD, standard deviation.

**Figure 2:**
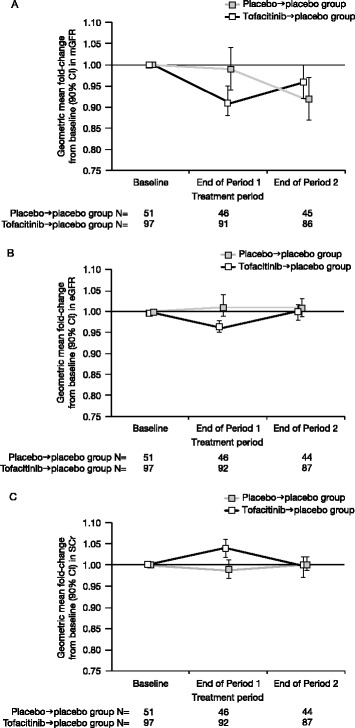
Adjusted geometric mean fold-change from baseline (90% CI) in measured glomerular filtration rate (mGFR) (A), estimated GFR (eGFR) (B) and serum creatinine (SCr) (C) (full analysis set, analysis of covariance, observed case).

Tofacitinib treatment in period 1 was associated with a reduction of 8% (90% CI: 2%, 14%) from baseline in adjusted geometric mean mGFR versus placebo. The reduction in geometric mean mGFR associated with tofacitinib in period 1 reversed during placebo treatment in period 2, and there was no statistically significant difference between the two treatment groups at the end of the study (ratio 1.04; 90% CI: 0.97, 1.11). The geometric mean fold-change in mGFR from baseline to the end of period 1 and period 2 is shown in Figure 2A. The sensitivity analysis results (the linear mixed-effects model on the FAS and ANCOVA from the PPAS) were comparable with the ANCOVA (FAS, observed case) analysis results.

Given the unexpected decline in mGFR in the placebo → placebo group in period 2, as well as the observation of outliers (high or low mGFR values that did not appear to correspond to SCr or eGFR for a given patient), a post-hoc analysis was performed after excluding the outlier patients (15 patients in the tofacitinib → placebo group and 9 patients in the placebo → placebo group). The results of the post-hoc analysis were consistent with results for the primary analyses but the significant decline in mGFR in period 2 for the placebo → placebo group was no longer apparent (geometric mean fold-change from baseline 0.98; 90% CI: 0.94, 1.01) (Table 3). A review of the outlier patients revealed no consistent demographic factors or concomitant medications, other than commonality of study centres.

**Table 3:** **Post-hoc analysis of mGFR change by excluding patients who had an outlier mGFR result (ANCOVA)**

	**mGFR (mL/min/1.73 m** ^**2**^ **)**	
	**Tofacitinib** → **placebo group**	**Placebo** → **placebo group**
**Change at end of period 1 from baseline**		
Number	76	37
Adjusted geometric mean fold-change (90% CI)	0.91 (0.88, 0.93)	1.01 (0.97, 1.04)
Ratio of adjusted geometric mean fold-change (90% CI)	0.90 (0.86, 0.94)	
**Change at end of period 2 from baseline**		
Number	72	36
Adjusted geometric mean fold-change (90% CI)	0.96 (0.93, 0.99)	0.98 (0.94, 1.01)
Ratio of adjusted geometric mean fold-change (90% CI)	0.98 (0.94, 1.03)	
**Change at end of period 2 from end of period 1**		
Number	72	36
Adjusted geometric mean fold-change (90% CI)	1.06 (1.03, 1.09)	0.99 (0.95, 1.03)
Ratio of adjusted geometric mean fold-change (90% CI)	1.07 (1.02, 1.12)	

Post-hoc analysis excluded those patients with a difference between mGFR and eGFR falling outside the 95% limits of agreement between the two methods of measurement. ANCOVA, analysis of covariance; eGFR, estimated glomerular filtration rate; mGFR, measured glomerular filtration rate.

**Figure 3:**
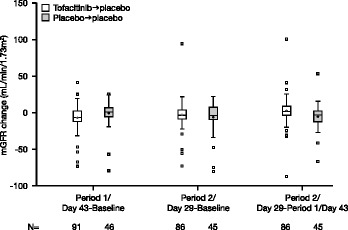
Box and whisker plot of change in measured glomerular filtration rate (mGFR) (full analysis set).

To more fully understand the variability in mGFR changes at the end of period 1 from baseline, an additional analysis of patients with greatest changes from the mean (defined by changes more than 1.5-times an interquartile distance from the upper and lower quartiles from the box plots (Figure 3)) was undertaken. Similar proportions in each treatment group fell into this category (six (6.6%) tofacitinib → placebo and three (6.5%) placebo → placebo patients). Of these, four tofacitinib → placebo and two placebo → placebo patients had decreases from baseline (mean of run-in and pre-dose day 1 mGFR measurements), and two tofacitinib → placebo patients and one placebo → placebo patient had increases from baseline in mGFR. In this analysis, mGFR measurements at the end of period 1 were similar to one of the components of the baseline for two patients. Additionally, mGFR tended to revert to a value within the range of the component baseline values at the end of period 2 for six patients. Increases in SCr were generally <0.06 mg/dL for this group of patients (maximum SCr changes of 0.12 and 0.13 mg/dL for the tofacitinib → placebo and placebo → placebo groups, respectively). Discontinuations (one patient for lack of efficacy and one patient for bronchopneumonia) occurred at least 15 days into period 2.

#### eGFR (Cockcroft-Gault)

Baseline eGFR was comparable between the two treatment groups (Table 2). In the tofacitinib → placebo group, median eGFR decreased from baseline to period 1 end and then increased following tofacitinib withdrawal in period 2 (Table 2). Overall, the median eGFR at period 2 end was similar compared with baseline (Table 2). In the placebo → placebo group, there was very little change in median eGFR between the three time points (Table 2).

At period 1 end, there was a 5% (90% CI: 2%, 8%) reduction from baseline in geometric mean eGFR associated with tofacitinib treatment versus placebo, which fully reversed during period 2 (geometric mean fold-change from end of period 1 to end of period 2, 1.03; 90% CI: 1.01, 1.05) and there was no difference between the two treatment groups at period 2 end (ratio 0.99; 90% CI: 0.97, 1.02) (Figure 2B). Unlike the mGFR, there was no change in the placebo → placebo group in either period 1 (geometric mean fold-change from baseline 1.01; 90% CI: 0.99, 1.04) or period 2 (geometric mean fold-change from end of period 1 to end of period 2, 1.00; 90% CI: 0.97, 1.02).

#### Serum creatinine

Baseline SCr was comparable between the two treatment groups (Table 2). At period 1 end there was a 5% (90% CI: 2%, 8%) increase from baseline in geometric mean SCr associated with tofacitinib treatment versus placebo. This SCr increase reversed during placebo treatment in period 2, and at period 2 end the treatment ratio was 1.01 (90% CI: 0.98, 1.04) (Figure 2C). No change was observed in SCr at the end of period 2 versus the end of period 1 for the placebo → placebo group.

### Efficacy endpoints

Patients receiving tofacitinib reported significantly greater improvements versus placebo in the efficacy endpoints measured, namely, ACR20/50/70 response rates, and least squares mean changes from baseline in DAS28-3(CRP) and DAS28-4(CRP).

### Safety

Treatment-emergent AEs (TEAEs) were reported in 42 and 26 patients in the tofacitinib → placebo and placebo → placebo groups, respectively. The majority of AEs were mild or moderate in intensity. The most frequently reported TEAEs were nasopharyngitis (n = 8 and n = 3), headache (n = 6 and n = 1), nausea (n = 5 and n = 2) and urinary tract infection (n = 3 and n = 2) in the tofacitinib → placebo and placebo → placebo groups, respectively.

One tofacitinib → placebo patient permanently discontinued during the placebo treatment period due to a serious AE (SAE) of bronchopneumonia. Four placebo → placebo patients experienced AEs resulting in permanent discontinuation: increased SCr (Additional file 1), somnolence, lymphopaenia and urticaria. Seven and zero patients temporarily discontinued their study drug or temporarily reduced their dose due to AEs in the tofacitinib → placebo and placebo → placebo groups, respectively; none of the AEs were serious. With the exception of an AE of pharyngitis, all AEs leading to temporary discontinuation or dose reduction occurred during period 1.

Two SAEs (pelvic fracture and bronchopneumonia) out of 97 patients occurred during period 2 in two tofacitinib → placebo patients. None of the 51 placebo → placebo patients had an SAE at any time. No patients experienced an AE coded to the narrow standardised MedDRA (Medical Dictionary for Regulatory Activities) query of ARF (including preferred terms of acute phosphate nephropathy, acute pre-renal failure, anuria, azotemia, continuous haemodiafiltration, dialysis, haemodialysis, nephropathy toxic, oliguria, peritoneal dialysis, renal failure, renal impairment). There were no deaths reported in this study.

No trends in clinically important treatment-related changes in vital signs, electrocardiogram or laboratory abnormalities were observed.

## Discussion

The results of this study suggest that mean increases in SCr and mean decreases in eGFR in patients with active RA treated with tofacitinib 10 mg BID occur in parallel with decreases in mGFR. Furthermore, these changes were reversible following withdrawal of tofacitinib 10-mg BID treatment; although mean mGFR did not return completely to baseline, there was no significant difference between tofacitinib and placebo groups at the end of the study. While this conclusion is complicated by the unexpected observations in the placebo → placebo group in period 2, it is further supported by the post-hoc analyses where the significant decline in the placebo → placebo group during period 2 was no longer evident. The mean changes in SCr and eGFR observed in this study are similar to those reported in phase-2 and phase-3 RA studies of tofacitinib.

An unexpected finding was a decrease in mean and median mGFR during the second period in placebo → placebo patients; as no concomitant changes in SCr were seen (the CIs for SCr changes were also much smaller than for mGFR), the interpretation and significance of the change in mGFR in the placebo → placebo group during this period remains unclear. Given the variability in mGFR and not in SCr, as well as the lack of agreement between the measurements for mGFR and SCr, it is likely that these unexpected findings in mGFR reflect measurement methodology issues rather than significant changes in GFR. Several studies have shown iohexol serum clearance to be highly correlated with the renal clearance of inulin, the gold standard of GFR measurement [22,24-27]; however, variability in mGFR measurements is expected given the inherently technical and complex nature of the iohexol serum clearance procedure [16,28]. This study required frequent and numerous timed blood samples to be collected and processed during four separate study visits in which the iohexol procedure was performed. Additionally, the procedure was conducted at multiple, global rheumatology study centres, rather than in a single clinical research unit, where patient diet, hydration and exercise could not be as rigidly controlled. Nonetheless, the variability observed in this study was consistent with the assumed variability used in the sample size calculation during protocol development and was within the range from previously published studies [10,29,30]. A decline in mGFR was also observed in placebo subjects in an earlier study investigating the effect of tofacitinib on renal function in healthy adult volunteers, hence, the importance of including a placebo group in the current mGFR study [10]. Post-hoc analyses were performed that excluded any mGFR outliers – those patients with likely aberrant mGFR results based on large discrepancies in mGFR and eGFR measurements. In the outlier analyses, the decline observed during period 2 for the primary analysis in the placebo → placebo group was not observed; however, the conclusions of the primary analyses that there was no significant difference in mGFR between tofacitinib and placebo groups at the end of the study was preserved. Therefore, despite the variability and potential technical limitations of the methodology, the study still detected a mean change in mGFR in patients with RA receiving tofacitinib.

Data on mGFR in patients with RA are limited. Changes in SCr and mGFR were not seen in a study of healthy volunteers dosed with tofacitinib, although study size and duration differences may not allow statements of differential effects in a specific population [10]. This study was not designed to address mechanisms for potential effects of tofacitinib on SCr or mGFR nor the relative impacts of inflammation and GFR changes on SCr; however, the reversibility of the effect, at least after short-term treatment, is reassuring, and supports the results of the SCr analyses across phase-2, phase-3 and LTE studies of tofacitinib in patients with RA [7]. In those studies, the relationship to baseline inflammation and its reduction, as well as to serum creatine kinase, suggests additional mechanisms may contribute to rises in SCr in patients receiving tofacitinib.

When applying to clinical practice, the changes in SCr and mGFR in tofacitinib-treated patients with RA should also be considered in the context of the concomitant medications received and the doses of such medications. It is unlikely that such changes in mGFR and SCr of the magnitude observed in this study will be clinically meaningful in the vast majority of patients; however, clinical judgement should be used when assessing changes in SCr or mGFR in patients with RA receiving tofacitinib. Data derived from a follow-up of patients on tofacitinib for up to 5 years in an LTE study do not suggest increased rates of renal AEs or progressive increases in levels of SCr [31], but ongoing assessment will further address long-term consequences.

## Conclusions

In summary, despite some potential operational limitations and technical challenges associated with the measurement of mGFR, the results of this study suggest that increases in SCr and decreases in eGFR in patients with RA treated with tofacitinib may reflect decreases in mGFR. Furthermore, changes in these parameters with short-term tofacitinib treatment appear reversible after discontinuation – mGFR returned towards baseline, with no significant difference from placebo at the end of the study, even after post-hoc analyses where the decline in mGFR in placebo → placebo patients was no longer present. The mechanisms behind these changes in renal endpoints are unknown. The overall safety and efficacy profiles were consistent with that of previous studies; safety monitoring will continue in ongoing and future clinical studies and routine pharmacovigilance to further assess the long-term and real-world renal safety profile of tofacitinib.

## Additional file

Additional file 1:
**Additional information, data and institutional review boards that are referred to within the main manuscript file.**

## Abbreviations

ACR: American College of Rheumatology
AE: adverse event
ANCOVA: analysis of covariance
ARF: acute renal failure
BID: twice daily
CI: confidence interval
CRP: C-reactive protein
DAS: disease activity score
DMARD: disease-modifying antirheumatic drug
eGFR: estimated glomerular filtration rate
FAS: full analysis set
GFR: glomerular filtration rate
HAQ-DI: health assessment questionnaire-disability index
IV: intravenous
JAK: Janus kinase
LTE: long-term extension
MedDRA: Medical Dictionary for Regulatory Activities
mGFR: measured glomerular filtration rate
NSAID: non-steroidal anti-inflammatory drug
PPAS: per-protocol analysis set
Q1: 25th percentile
Q3: 75th percentile
RA: rheumatoid arthritis
SAE: serious adverse event
SCr: serum creatinine
SD: standard deviation
TEAE: treatment-emergent adverse event

## References

[1] Burmester GR, Blanco R, Charles-Schoeman C, Wollenhaupt J, Zerbini C, Benda B (2013). Tofacitinib (CP-690,550) in combination with methotrexate in patients with active rheumatoid arthritis with an inadequate response to tumour necrosis factor inhibitors: a randomised phase 3 trial. Lancet..

[2] Fleischmann R, Kremer J, Cush J, Schulze-Koops H, Connell CA, Bradley JD (2012). Placebo-controlled trial of tofacitinib monotherapy in rheumatoid arthritis. N Engl J Med..

[3] Kremer J, Li ZG, Hall S, Fleischmann R, Genovese M, Martin-Mola E (2013). Tofacitinib in combination with nonbiologic DMARDs in patients with active rheumatoid arthritis: a randomized trial. Ann Intern Med..

[4] van der Heijde D, Tanaka Y, Fleischmann R, Keystone E, Kremer J, Zerbini C (2013). Tofacitinib (CP-690,550) in patients with rheumatoid arthritis receiving methotrexate: twelve-month data from a twenty-four-month phase III randomized radiographic study. Arthritis Rheum..

[5] van Vollenhoven RF, Fleischmann R, Cohen S, Lee EB, García Meijide JA, Wagner S (2012). Tofacitinib or adalimumab versus placebo in rheumatoid arthritis. N Engl J Med..

[6] Lee EB, Fleischmann R, Hall S, Wilkinson B, Bradley J, Gruben D (2014). Tofacitinib versus methotrexate in rheumatoid arthritis. N Engl J Med..

[7] Isaacs J, Zuckerman A, Krishnaswami S, Nduaka C, Lan S, Hutmacher M (2014). Changes in serum creatinine in patients with active rheumatoid arthritis treated with tofacitinib: results from clinical trials. Arthritis Res Ther..

[8] European Medicines Agency CfMPfHU. European Public Assessment Report for Xeljanz, International non-proprietary name: tofacitinib. http://www.ema.europa.eu/docs/en_GB/document_library/EPAR_-_Public_assessment_report/human/002542/WC500154697.pdf.

[9] US Food and Drug Administration CfDEaR. Pharmacology review of NDA, Xeljanz (tofacitinib). http://www.accessdata.fda.gov/drugsatfda_docs/nda/2012/203214Orig1s000PharmR.pdf.

[10] Lawendy N, Krishnaswami S, Wang R, Gruben D, Cannon C, Swan S (2009). Effect of CP-690,550, an orally active janus kinase inhibitor, on renal function in healthy adult volunteers. J Clin Pharmacol..

[11] Kimura N, Masuda S, Tanihara Y, Ueo H, Okuda M, Katsura T (2005). Metformin is a superior substrate for renal organic cation transporter OCT2 rather than hepatic OCT1. Drug Metab Pharmacokinet..

[12] Klamerus KJ, Alvey C, Li L, Feng B, Wang R, Kaplan I (2014). Evaluation of the potential interaction between tofacitinib and drugs that undergo renal tubular secretion using metformin, an in vivo marker of renal organic cation transporter 2. Clinical Pharm in Drug Dev..

[13] Karstila K, Harmoinen AP, Lehtimaki TJ, Korpela MM, Mustonen JT, Saha HH (2008). Measurement of the kidney function in patients with rheumatoid arthritis: plasma cystatin C versus 51Cr-EDTA clearance. Nephron Clin Pract..

[14] Perrone RD, Madias NE, Levey AS (1992). Serum creatinine as an index of renal function: new insights into old concepts. Clin Chem..

[15] Stevens LA, Coresh J, Greene T, Levey AS (2006). Assessing kidney function–measured and estimated glomerular filtration rate. N Engl J Med..

[16] Stevens LA, Levey AS (2009). Measured GFR as a confirmatory test for estimated GFR. J Am Soc Nephrol..

[17] Dougados M, Soubrier M, Antunez A, Balint P, Balsa A, Buch MH (2014). Prevalence of comorbidities in rheumatoid arthritis and evaluation of their monitoring: results of an international, cross-sectional study (COMORA). Ann Rheum Dis..

[18] McInnes I, Schett G (2011). The pathogenesis of rheumatoid arthritis. N Engl J Med..

[19] Michaud K, Wolfe F (2007). Comorbidities in rheumatoid arthritis. Best Pract Res Clin Rheumatol..

[20] Strand V, Khanna D (2010). The impact of rheumatoid arthritis and treatment on patients’ lives. Clin Exp Rheumatol..

[21] Arnett FC, Edworthy SM, Bloch DA, McShane DJ, Fries JF, Cooper NS (1988). The American Rheumatism Association 1987 revised criteria for the classification of rheumatoid arthritis. Arthritis Rheum..

[22] Gaspari F, Perico N, Ruggenenti P, Mosconi L, Amuchastegui CS, Guerini E (1995). Plasma clearance of nonradioactive iohexol as a measure of glomerular filtration rate. J Am Soc Nephrol..

[23] Schwartz GJ, Work DF (2009). Measurement and estimation of GFR in children and adolescents. Clin J Am Soc Nephrol..

[24] Brown SC, O’Reilly PH (1991). Iohexol clearance for the determination of glomerular filtration rate in clinical practice: evidence for a new gold standard. J Urol..

[25] Erley CM, Bader BD, Berger ED, Vochazer A, Jorzik JJ, Dietz K (2001). Plasma clearance of iodine contrast media as a measure of glomerular filtration rate in critically ill patients. Crit Care Med..

[26] Olsson B, Aulie A, Sveen K, Andrew E (1983). Human pharmacokinetics of iohexol. A new nonionic contrast medium. Invest Radiol.

[27] Rahn KH, Heidenreich S, Bruckner D (1999). How to assess glomerular function and damage in humans. J Hypertens..

[28] Hsu CY, Bansal N (2011). Measured GFR as “gold standard”–all that glitters is not gold?. Clin J Am Soc Nephrol..

[29] Vincenti F, Tedesco SH, Busque S, O’Connell P, Friedewald J, Cibrik D (2012). Randomized phase 2b trial of tofacitinib (CP-690,550) in de novo kidney transplant patients: efficacy, renal function and safety at 1 year. Am J Transplant..

[30] Gaspari F, Perico N, Matalone M, Signorini O, Azzollini N, Mister M (1998). Precision of plasma clearance of iohexol for estimation of GFR in patients with renal disease. J Am Soc Nephrol..

[31] Wollenhaupt J, Silverfield J, Lee EB, Curtis JR, Wood SP, Soma K (2014). Safety and efficacy of tofacitinib, an oral Janus kinase inhibitor, for the treatment of rheumatoid arthritis in open-label, longterm extension studies. J Rheumatol..

