# Supplementary material for: Evaluation of the effect of tofacitinib on measured glomerular filtration rate in patients with active rheumatoid arthritis: results from a randomised controlled trial
Source: Arthritis Res Ther. 2015 Apr 6;17(1):95. doi: 10.1186/s13075-015-0612-7 (PMC4445792; doi:10.1186/s13075-015-0612-7)
Supplement: Additional file 1: — Additional information, data and institutional review boards that are referred to within the main manuscript file. [file 13075_2015_612_MOESM1_ESM.docx]

**Supplementary Section**

***Protocol amendments***

Updates were made to lymphocyte count exclusion criteria in the August 2012 Investigator’s Brochure. Furthermore, the following amendments were made to incorporate changes noted in the Administrative Change Letter issued 15 February 2012: addition of screening visit laboratory tests for hepatic transaminase laboratory parameters for patients starting isoniazid treatment and Hepatitis B core antibody testing; clarified the use of topical medications and what is considered highly effective methods of contraception; minor editorial changes to ensure consistency between protocol sections.

***Iohexol serum clearance***

The iohexol dose was 20 mL (647 mg iohexol/mL, equivalent to 300 mg iodine/mL). A pre‑dose blood sample was collected then the morning dose of study medication was administered immediately prior to the iohexol bolus. Blood samples were collected at 10, 20, 30, 60, 120, 240 and 300 minutes after the start time of the iohexol IV bolus. All mGFR evaluations were performed in the morning. Patients were instructed to maintain their usual diet (including diet composition and sodium intake) and activity level from the run-in period through the end of Period 2. Prior to the mGFR evaluation and following mGFR evaluation, patients should have had adequate fluid intake (≥2000 mL). During mGFR evaluation, patients should have drunk approximately 200 mL of water per hour through 300 min after administration of iohexol.

***Sample size determination***

Based on the assumption of a common standard deviation of 0.28 for both treatment groups for the change in the natural log‑transformed mGFR (mL/min), a sample size of 120 patients (80 for tofacitinib and 40 for placebo) would have approximately 90% probability that the observed half‑width of the 2-sided 90% CI for the ratio between the two treatment groups (tofacitinib→placebo/placebo→placebo) in the geometric mean fold change of mGFR from baseline to the end of Period 1 was within 10% of the observed ratio. Assuming a dropout rate of 10% during Period 1, approximately 132 patients (88 in tofacitinib and 44 in placebo groups) were to be randomised.

***Patient narrative for increased SCr***

The patient in the placebo→placebo treatment group who permanently discontinued due to increased SCr experienced a SCr increase to 1.48 mg/dL on Day 22 (reference range:
0.45–1.35 mg/dL) that was of mild severity. This patient had SCr levels of 0.87, 0.85, 1.48, 1.59, 1.28, and 1.21 at screening (Day -14), baseline (Day 1), Day 22, Day 29, Day 37 and Day 49, respectively. The last dose of study drug was on Day 30; the event was considered resolved on Day 65 and was considered by the investigator to be treatment-related.

***List of Institutional Review Boards***

1. Eticka komise IKEM a TN, Videnska 800, Praha 4 Krc, 14059, Czech Republic
2. Ethik-Kommission der Medizinischen Fakultaet der Friedrich-Alexander-Universitaet, Erlangen-Nuernberg, Krankenhausstrasse 12, Erlangen, 91054, Germany
3. Severance Hospital, Yonsei University Health System, Institutional Review Board Severance Hospital, Yonsei University Health System, 250 Seongsanno, Seodaemun-Gu, Seoul, 120-752, Korea, Republic Of
4. Comite de Etica e Investigacion en Salud (CEIS), Unidad de Investigacion Clinica del
5. Centro de Especialidades Medicas del Sureste S.A. de C.V., Calle 60 No. 329-B entre 35 y Av., Colon, Col. Centro, Merida, Yucatan 97000, Mexico
6. Komisja Bioetyczna Uniwersytetu, Mikolaja Kopernika w Toruniu, przy Collegium Medicum im., Ludwika Rydygiera w Bydgoszczy, ul. Marii Sklodowskiej Curie 9, Bydgoszcz, 85-094, Poland
7. Local Ethics Committee at the Almazov Federal Heart, Blood and Endocrinology Centre, 15 Prospect Parkhomenko, Saint Petersburg, 194156, Russian Federation
8. Local Ethics Committee at the L.G. Sokolov Clinical Hospital #122, 4 Prospect Kultury, Saint-Petersburg, 194291, Russian Federation
9. Ethics Committee at the Republican Hospital n.a. V.A. Baranov, Pirogova str., 3, Petrozavodsk, 185019, Russian Federation
10. Ethics Committee at Research, Institute of Rheumatology, 34 A Kashirskoe shosse, Moscow, 115522, Russian Federation
11. Comite Etico de Investigacion Clinica. Comunidad Autonoma del Pais Vasco (CEIC-E), Direccion de Farmacia, Departamento de Sanidad del Gobierno Vasco, Secretaria Tecnica CEIC-E, C/ Donostia-San Sebastian, nº 1Vitoria, Alava 01010, Spain
12. Quorum Review Institutional Review, Board Incorporated Suite 1000, 1601 Fifth Avenue, Seattle, WA 98101, United States
